# Spatial heterogeneity as the structure feature for structure–property relationship of metallic glasses

**DOI:** 10.1038/s41467-018-06476-8

**Published:** 2018-09-27

**Authors:** Fan Zhu, Shuangxi Song, Kolan Madhav Reddy, Akihiko Hirata, Mingwei Chen

**Affiliations:** 1 State Key Laboratory of Metal Matrix Composites, School of Materials Science and Engineering, Shanghai Jiao Tong University, 200030 Shanghai, China; 2 WPI Advanced Institute for Materials Research, Tohoku University, 980-8577 Sendai, Japan; 3 Department of Materials Science and Engineering, Johns Hopkins University, Baltimore, MD 21214 USA

## Abstract

The mechanical properties of crystalline materials can be quantitatively described by crystal defects of solute atoms, dislocations, twins, and grain boundaries with the models of solid solution strengthening, Taylor strain hardening and Hall–Petch grain boundary strengthening. However, for metallic glasses, a well-defined structure feature which dominates the mechanical properties of the disordered materials is still missing. Here, we report that nanoscale spatial heterogeneity is the inherent structural feature of metallic glasses. It has an intrinsic correlation with the strength and deformation behavior. The strength and Young’s modulus of metallic glasses can be defined by the function of the square root reciprocal of the characteristic length of the spatial heterogeneity. Moreover, the stretching exponent of time-dependent strain relaxation can be quantitatively described by the characteristic length. Our study provides compelling evidence that the spatial heterogeneity is a feasible structural indicator for portraying mechanical properties of metallic glasses.

## Abstract (web summary)

Directly relating the mechanical properties of metallic glasses to their atomic structure remains a challenge. Here, the authors use high resolution microscopy to show many mechanical properties of metallic glasses depend on a single structural parameter, the characteristic length of spatial heterogeneity.

## Introduction

Owing to the absence of periodic structures and hence dislocation like defects, a well-defined structure–property relationship for metallic glasses has not been established^1–6^. To describe the mechanical behaviors of metallic glasses, free volumes^7^ and shear transformation zones (STZs)^8–11^ have been introduced to depict the structure instability during elastic-to-plastic transition of metallic glasses. Although the STZ theory is based on the assumption of structural heterogeneity in metallic glasses as structural origins of shear localization and shear softening, a distinct relationship between the structural inhomogeneity and macroscopic mechanical properties of metallic glasses has not been established. Extensive computational simulations^12–15^ and experimental characterizations^16–19^ have demonstrated that the structural inhomogeneity of metallic glasses is associated with nanoscale structure fluctuation, which possibly inherits from spatially heterogeneous dynamics in low-temperature supercooled liquids prior to glass transition. The assumption of soft or liquid-like regions in spatial heterogeneity has been widely used to account for the anelastic deformation^20–26^ and the structural rejuvenation^27–29^ of metallic glasses. However, a quantitative relationship between spatial heterogeneity and macroscopic mechanical properties of metallic glasses has not been experimentally determined and, thus, the intrinsic correlation between spatial heterogeneity and properties remains unknown. This is partially because of the technical challenges in quantitatively measuring and describing the structural feature of spatial heterogeneity^30,31^.

In the context of our recent success in experimentally characterizing the spatial heterogeneity of metallic glasses by amplitude-modulation dynamic atomic force microscopy (AM-AFM) and scanning transmission electron microscopy (STEM)^18,31,32^, in this study, we systematically investigate the relationship between macroscopic mechanical properties and the characteristic length of spatial heterogeneity of metallic glasses, and demonstrate that spatial heterogeneity is a distinguishing structural feature of metallic glasses for describing the structure–property relationship of disordered materials.

## Results

### Characterization of spatial heterogeneity

**Fig. 1:**
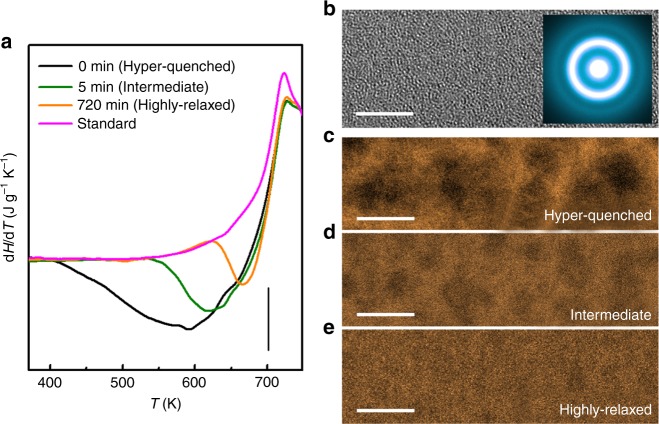
Spatial heterogeneity of metallic glasses with different thermodynamic statuses. **a** Heat flow traces of the as-prepared hyper-quenched sample and the samples annealed at 553 K (~0.8*T*_g_) for 5 min (intermediate) and 720 min (highly relaxed). The heat flow trace of a standard samples is also plotted for reference. Scale bar, 0.2. **b** High-resolution TEM (HRTEM) image of the hyper-quenched metallic glass. The inset is the corresponding selected area electron diffraction pattern. Scale bar, 5 nm. High-angle annular dark-field scanning TEM (HAADF-STEM) images of **c** the hyper-quenched, **d** the intermediate and **e** the highly relaxed samples. Scale bar, 5 nm

To facilitate the measurements of spatial heterogeneity, a hyper-quenched Zr_53_Cu_36_Al_11_ (at%) metallic glass with a large excess enthalpy below the glass transition temperature *T*_g_ (~695 K) is prepared as shown in Fig. 1a. A series of hyper-quenched samples are annealed at 553 K (~0.8*T*_g_) for different durations from 5, 30 and 180 min to 720 min to tune the thermodynamic status and structure of the metallic glass. During the annealing, the excess enthalpy below *T*_g_ is successively released. The nominal cooling rate for the hyper-quenched sample is estimated to be ∼2.4 × 10^7^ K s^−1^ according to the scaled Arrhenius plot of fictive temperatures versus cooling rates^33^. After the sub-*T*_g_ annealing for 5 and 720 min, the nominal cooling rates of the samples decrease to ~3.3 × 10^3^ and ~12 K s^−1^, respectively. Hereafter, the samples annealed for 5 and 720 min are termed the intermediate and the highly relaxed samples, respectively. The hyper-quenched sample without annealing is inspected by high-resolution TEM (HRTEM) and a uniform maze-like structure of amorphous alloys can be seen (Fig. 1b). The diffraction halo in the inset further confirms that the hyper-quenched metallic glass is fully amorphous. The intermediate and the highly relaxed samples are also inspected by HRTEM and verified to be fully amorphous without visible contrast variation. However, when the samples are imaged by high-angle annular dark-field STEM (HAADF-STEM), inhomogeneous contrast with dark and bright regions can be seen. The dark regions have a size of ∼6 nm and are evenly distributed in the hyper-quenched sample as shown in Fig. 1c. The dark regions become indistinguishable after annealing and the characteristic size gradually decreases with the extension of annealing time as shown in Fig. 1d and e. After annealing for 720 min at 553 K, the size of the dark regions degenerates to about 2.8 nm. On the basis of our previous results, the dark regions correspond to the regions with relatively low density and loosely packed atomic structure in spatial heterogeneity^32^, which are expected to be responsible to the initiation of inelastic deformation and sub-*T*_g_ relaxation^18^. The sizes of dark regions estimated from HAADF-STEM images are in a good agreement with the characteristic lengths of spatial heterogeneity measured by AM-AFM.

### Instrumented nanoindentation

**Fig. 2:**
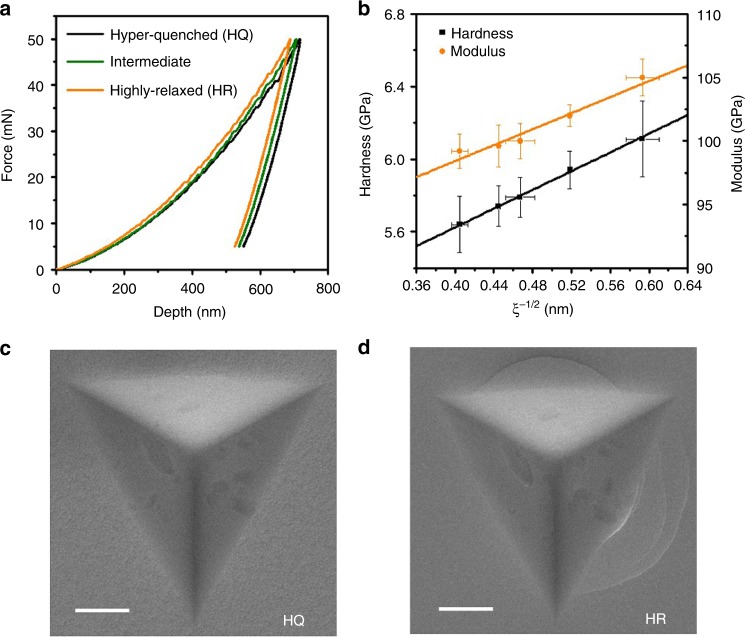
Mechanical property measurements by instrumented nanoindentation. **a** Nanoindentation force–depth curves of the hyper-quenched, the intermediate and the highly relaxed samples. **b** Nanoindentation hardness and modulus plotted with the characteristic length of spatial heterogeneity. The solid curve is a linear fitting for the points. The indenter impressions of **c** the hyper-quenched, and **d** the highly relaxed samples. Scale bar, 1 μm. The error bars indicate standard deviation

An instrumented nanoindentation is employed to measure the mechanical properties of the samples with different characteristic lengths of spatial heterogeneity. The nanoindentation force–depth curves are shown in Fig. 2a. To minimize the indenter size effect on hardness, a large load of 50 mN is used with the penetration depth of ∼600–700 nm, far beyond the strong depth sensitive region of ∼300 nm (Supplementary Fig. 1). The penetration depth of the indenter becomes smaller with the decrease in the characteristic length. The hardness *H* and Young’s modulus *M* are calculated according to the Olive–Pharr method^34^. For the hyper-quenched sample, *H* and *M* are measured to be 5.64 ± 0.155 and 99.2 ± 1.37 GPa, respectively. Both hardness and modulus increase with the degeneration of spatial heterogeneity. For the highly relaxed sample, *H* and *M* are as high as 6.11 ± 0.209 and 105 ± 1.47 GPa, respectively. The hardness and modulus of the samples are plotted as a function of the characteristic length *ξ* (Fig. 2b). Surprisingly, it is found that the relationships of *ξ* with hardness *H* and modulus *M* can be best fitted by the equations: $H = 4.59{\mathrm{ + 2}}{\mathrm{.58}}\xi ^{ - 1/2}$ and $M = 85.8{\mathrm{ + 31}}{\mathrm{.5}}\xi ^{ - 1/2}$, the well-known Hall–Petch relation of polycrystalline materials. When *ξ* is infinite, the hardness is ~4.59 GPa and the modulus is ~85.8 GPa, which may correspond to the strength of the extremely disordered metallic glass with the maximum excess enthalpy. When we assume that the *ξ* is equal to the average diameter of the short-range order (~0.316 nm, i.e. the approximate size of a Zr-centered cluster with the nearest neighbors), the hardness and modulus are estimated to be ∼9.18 and ∼142  GPa, respectively. These values are very close to those of ultrastable metallic glass^35^. Additionally, no remarkable shear bands can be seen around the impression of the soft hyper-quenched sample (Fig. 2c), while several shear bands are visible from the highly relaxed sample (Fig. 2d), indicating a possible transition of deformation mode from relatively homogeneous flow to localized shearing owing to the degeneration of spatial heterogeneity^36,37^.

### Micro-pillar compression

**Fig. 3:**
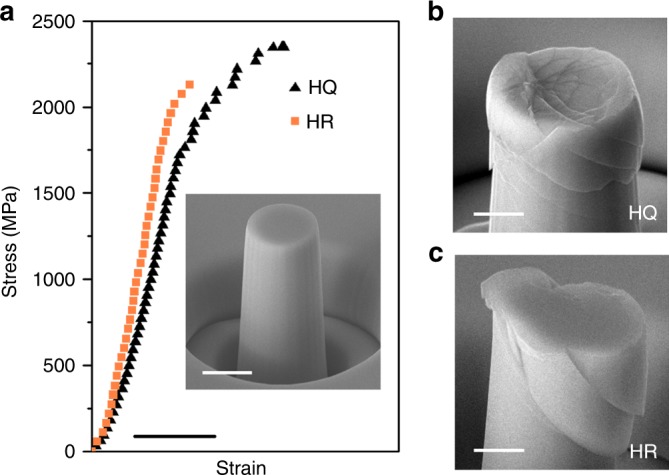
Micro-pillar compression testing. **a** Engineering stress–stain curves of the hyper-quenched and the highly relaxed samples subjected to the uniaxial micro-pillar compression. Scale bar, 2%. The micro-pillars after deformation for **b** the hyper-quenched and **c** the highly relaxed samples. Scale bar, 1 μm. The inset is a micro-pillar before compression. Scale bar, 2 μm

The correlations of spatial heterogeneity with mechanical properties and plastic deformation are also investigated by micro-pillar compression testing. Figure 3a presents the engineering stress–strain curves of the hyper-quenched and highly relaxed samples tested at room temperature. The yielding strength and Young’s modulus of the hyper-quenched sample are measured to be around 1810 MPa and 92.0 GPa, respectively. With the degeneration of spatial heterogeneity, the yielding strength and Young’s modulus of the highly relaxed sample increase to 2050 MPa and 109 GPa, respectively. The ratios between the nanoindentation hardness and the yielding strength for both samples are about 3.0, which is in accord with the empirical value for metallic glasses^38^. Although the shape of micro-pillars and the alignment of loading can affect the accuracy of the mechanical properties, the measured strength and modulus have a good agreement with the nanoindentation measurements. In addition, the hyper-quenched sample exhibits a work-hardening-like deformation together with profuse tiny shear bands (Fig. 3b), while the highly relaxed sample fails right after the yielding in a brittle manner along a primary shear band (Fig. 3c). The differences in deformation and failure modes under the uniaxial loading condition further demonstrate that the spatial heterogeneity has strong correlations with mechanical properties and plastic deformation of metallic glasses.

### Strain relaxation

**Fig. 4:**
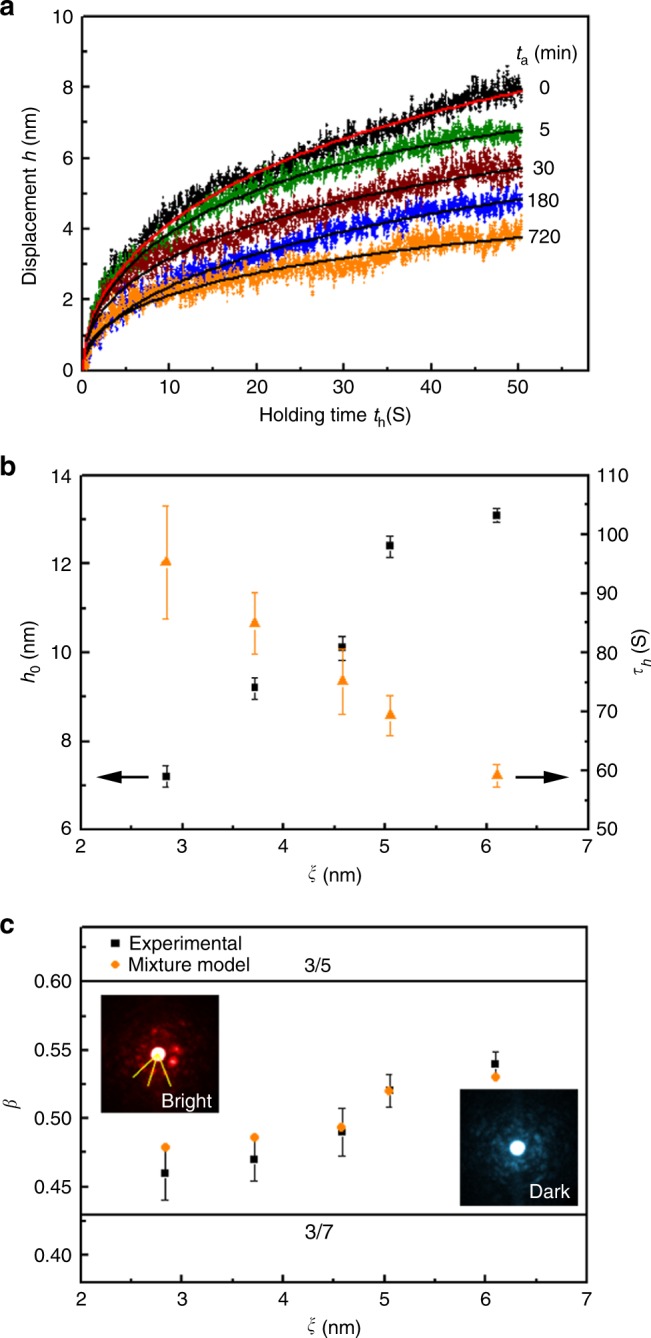
Strain relaxation measurements. **a** Indenter displacement *h* as a function of the holding time *t*_h_ at a constant loading of 50 mN. The solid lines are fitting curves of the raw data. **b** The relaxation amplitude *h*_0_ and the characteristic relaxation time *τ*_h_ versus the characteristic length *ξ* of spatial heterogeneity. **c** The stretching exponent *β* plotted with the characteristic length *ξ*. The experimental values of *β* measured by the strain relaxation are well consistent with the predictions of the mixture model. The top left inset is a representative ABED pattern for the bright regions, and the bottom right inset for the dark regions. The error bars indicate standard deviation

Room-temperature strain relaxation by nanoindentation is carried out to investigate the influence of spatial heterogeneity on the time-dependent deformation of metallic glasses. The maximum loading of 50 mN is applied on the samples in 1 s, held for 50 s for strain relaxation, and then unloaded in 1 s. The evolution of displacement *h* in the holding segment with holding time *t*_h_ is presented in Fig. 4a. Although the applied force is kept constant at 50 mN, the indenter tip gradually penetrates into the hyper-quenched sample as deep as 8 nm within 50 s, indicating that time-dependent deformation takes place at room temperature. Moreover, the penetrating rate progressively slows down and the displacement reaches a stationary value. After annealing, the maximum penetration displacement gradually decreases to less than 4 nm for the highly relaxed sample. A KWW-type function $h = h_0\left[ {1 - {\mathrm{exp}}( - (t_{\mathrm{h}}/\tau _{\mathrm{h}})^\beta )} \right]$ is applied to fit the relaxation curves, where *h*_0_ is the amplitude of displacement, *τ*_h_ the characteristic relaxation time and *β* the stretching exponent. We plot *h*_0_ and *τ*_h_ of each sample with the corresponding characteristic length of spatial heterogeneity in Fig. 4b. The displacement amplitude increases from 7.2 to 13.1 nm along with the increase in characteristic length from ~2.8 to ~6.1 nm. Meanwhile, the characteristic relaxation time becomes much shorter from 95.3 to 59.2 s.

Figure 4c plots the stretching exponent *β* as a function of the characteristic length of spatial heterogeneity. The value of *β* shows a positive relationship with the characteristic length of spatial heterogeneity and gradually increases from 0.46 for the highly relaxed sample to 0.54 for the hyper-quenched metallic glass. This is consistent with the general description that *β* is an indicator of structural and dynamic heterogeneity of supercooled liquids and glasses^39,40^. In particular, we note that these stretching exponents are between two distinctive numbers 3/5 and 3/7^41,42^. The bifurcation of the stretching exponents for glass relaxation into two values, 3/5 (or 0.6) and 3/7 (or 0.43) has been observed from a wide range of glasses. The two numbers can be derived based on the diffusion-trap model where it assumes that the glass relaxation depends on the effective dimensionality of the channels along which the relaxation can proceed^41^. According to this model, the stretching exponent is derived as *β* = *d*/(*d* + 2), where *d* is the effective dimensionality of relaxation channels. When all the channels are activated in three-dimensional space, *d* equals to 3 and, then, one can obtain *β* = 3/5. When only the long-range part of the channels are activated with a fraction of 1/2, *d* equals to 3/2 and one can obtain *β* = 3/7. The transition of the stretching exponents from 0.54 to 0.46 observed in our experiment implies a more fractal dimensionality related to the degeneration of the spatial heterogeneity. To elucidate the structural origins of the changes in the dimensionality, we analyze the local atomic order of spatial heterogeneity by angstrom-beam electron diffraction (ABED) based on our previous observations^32,43^. As explicated by a representative ABED pattern from the bright regions (the top left inset in Fig. 4c), the local atomic packing contains icosahedron-like short-range order with a relatively dense packing, while a representative ABED pattern from the dark regions (the bottom right inset in Fig. 4c) shows a more disordered structure with very weak on-phase scattering from the nearest neighbors. Thus, the atoms in dark regions are loosely packed and have more free space to move in three dimensions. If a metallic glass is composed of only dark regions, the stretching exponent will be 3/5 (0.6) slightly larger than the *β* = 0.54 for the hyper-quenched metallic glass. With the degeneration of spatial heterogeneity by annealing, the icosahedral order becomes more pronounced with the increase of bright regions. Therefore, the mobility of atoms in metallic glass is more and more restricted by the increasing local atomic ordering. Additionally, we notice that the icosahedral order in the expanded bright regions becomes more frustrated as the angular distribution of ABED is more deviated from the value of perfect icosahedron (Supplementary Figs. 2 and 3). The icosahedral order can be regarded as a 5D space structure constituted of a real space with a dimensionality *d*_1_ = 3 and a complementary phason space with a dimensionality *d*_2_ = 2^44^. The effective dimensionality can be calculated to be *d* = 3*d*_1_/(*d*_1_ + *d*_2_) = 1.8, corresponding to *β* = 0.474 which is very close to 0.46 for the highly relaxed sample in our measurement. Therefore, we assume that the metallic glass is a simple mixture of dark and bright regions and the relaxation behavior of the whole sample is contributed simultaneously by the two regions. Accordingly, the stretching exponent can be estimated by a linear composition model: *β* = 0.6*V*_D_ + 0.474*V*_B_, where *V*_D_ is the volume fraction of dark regions and *V*_B_ the bright regions. Since the total volume fraction of *V*_D_ and *V*_B_ equals to 1, the above equation can be simplified as *β* = 0.474 + 0.126*V*_D_. The stretching exponent *β* for each sample is calculated according to the volume fraction of dark regions in the spatial heterogeneity. Based on the HAADF-STEM image, *V*_D_ for the hyper-quenched metallic glass is estimated to be around 0.44, and the stretching exponent is then calculated to be about 0.53, very close to the experimental value *β* = 0.54. For the sample annealed for 5 min, *V*_D_ is estimated from the HAADF-STEM image, and for other samples the fractions are assessed based on the AM-AFM images. The calculated stretching exponents for each sample on the basis of the experimentally measured *V*_D_ are plotted and compared with the experimental ones as shown in Fig. 4c. The good coincidence between the calculated and the experimental values proves that the strain relaxation of metallic glasses is determined by the relative volumes of two regions of spatial heterogeneity.

## Discussion

The quantitative expression of the strain relaxation based on the effective dimensionality of icosahedral order explicitly suggests that the mechanical behaviors of metallic glasses are strongly correlated with the local atomic order of metallic glasses. Although the percolation of stable structure has long been assumed to contribute to the stiffness of metallic glass^14^, direct experimental evidence has not been obtained. Our recent investigation on spatial heterogeneity utilizing ABED has determined that the bright regions are composed of pronounced icosahedral order, while the dark regions are more structurally disordered^32^. According to the scenario of geometric frustration^45–48^, the five-fold symmetry of icosahedral order cannot extend to completely fill a three-dimensional space and only form the percolation of icosahedral order. The remaining spaces may become the dark regions with a relatively loose atomic packing. Our previous AM-AFM measurements^18,31^ has demonstrated that the dark regions in spatial heterogeneity have a lower viscoelasticity and can be stimulated at low stresses, which might initiate STZs for plastic deformation and thus control the strength. As revealed by our previous ABED experiments and MD simulations^32^, in the Zr_53_Cu_36_Al_11_ (at%) metallic glass the icosahedral order in bright regions is mainly contributed by Cu-centered and Al-centered short-range order, while the loosely packed dark regions are usually dominated by Zr-centered short-range order. According to a recent high-pressure extended x-ray absorption fine structure (EXAFS) study^49^, the Zr–Zr pairs will be strained preferentially during deformation and the tight Zr–Cu pairs will contribute to the stiffness. Upon a large strain, the Zr–Cu pairs may be involved into the deformation and result in increased nonaffine displacements and eventually the formation of STZs and shear bands^50^.

The spatial heterogeneity is most likely inherited from the dynamic heterogeneity of supercooled liquids in which slow and fast dynamic domains with nanoscale size are frozen during the glass transition^16^. In principle, the spatial heterogeneity represents the structure and kinetic state of supercooled liquids at the temperature where the dynamics of supercooled liquids are arrested. The sub-*T*_g_ annealing is equivalent to slowly cooling the supercooled liquid down to a lower temperature associated with an expansion of bright regions of spatial heterogeneity. Apparently, the length scale of spatial heterogeneity is far beyond that described by conventional pair distribution function (PDF) which is more sensitive to short-range and medium-range order. In fact, the nano-scale spatial heterogeneity is usually hidden in the width of PDF peaks. The freshly quenched metallic glasses with significant dark regions of spatial heterogeneity have a broader PDF peaks because of the larger variation in interatomic distance.

The Hall–Petch relation between hardness/modulus and the characteristic length also implies that the stress-driven transition from elastic to inelastic deformation is determined by the characteristic length of spatial heterogeneity. Although the Hall–Petch relation was originally proposed for the strengthening induced by the interaction between dislocations and grain boundaries in polycrystalline materials, the square root reciprocal function has been found to work well for the strengthening effect of various types of boundaries introduced by plastic deformation beyond dislocation plasticity^51,52^. For example, the strength of brittle ceramics, which do not experience any dislocation deformation, also follows the Hall–Petch relation. For the observed square root reciprocal relation between the strength and the characteristic length of spatial heterogeneity in metallic glasses, the underlying mechanisms have not been well understood. It is most likely related to the local modulus variations and the activation of STZs from dark regions. In particular, the planar feature of shear bands, which is initiated by the assembly of a critical number of STZs and responsible to the yielding and deformation, is similar to the planar slip of dislocations in crystals and may lead to the square root reciprocal relation. The larger characteristic length may require fewer activated dark regions for cooperatively initiating a shear band at a lower stress level. Simultaneously, the densely packed bright regions work as the backbone for the stiffness of metallic glass, and thus the length dependence of modulus might simply originate from the relative fraction of dark and bright regions of spatial heterogeneity. More experimental and theoretical investigations are required for a comprehensive understanding of the quantitative size dependence of mechanical properties in the disordered materials.

In summary, we systematically investigate the relation between spatial heterogeneity and mechanical behaviors of metallic glasses. The strength, modulus, and relaxation exponent of metallic glasses can be quantitatively expressed by the characteristic length of spatial heterogeneity. Although the underlying mechanisms of both stretching exponent and Hall–Petch relation, discovered by this study are not clear and warrant future studies, this work provides a well-defined structural feature to describe the mechanical properties of metallic glasses, which will be important in understanding the structural origins of mechanical behaviors of metallic glasses and in designing novel metallic glasses with improved properties.

## Methods

### Sample preparation

Hyper-quenched metallic glass samples with a composition of Zr_53_Cu_36_Al_11_ (at%) are deposited on Si substrates at ~0.2 nm s^−1^ by RF magnetron sputtering at room temperature. The sub-*T*_g_ annealing of the hyper-quenched metallic glass is conducted in a Pt furnace under the protection of flowing Ar gas. The heat flow traces are measured by a differential scanning calorimetry (DSC, Perkin-Elmer 8500) at a heating rate of 20 K min^−1^. The standard sample is prepared by slowly cooling the hyper-quenched sample from its supercooled liquid region at 20 K min^−1^ and then subjected to the second up-scan to obtain the standard heat flow trace.

### Structural characterization

TEM measurements are performed by a Cs-corrected TEM (JEOL JEM-2100F). The ABED pattern for one individual atomic configuration in metallic glass is acquired by utilizing a nearly parallel electron beam with a full width at half maximum (FWHM) diameter of 0.36 nm. TEM samples are carefully prepared by ion milling with 3 keV Ar ions cooled by liquid nitrogen. An AM-AFM (Bruker MultiMode) equipped with a pyramidal silicon tip with a sharp diamond-like spike of ~1 nm is applied to characterize the spatial heterogeneity in metallic glasses.

### Instrumented nanoindentation

An instrumented nanoindenter (MTS G200) equipped with a Berkovich tip is used to measure the hardness and modulus of samples with a maximum loading of 50 mN and a loading rate of 0.1 mN  s^−1^ at room temperature. Twenty-five points are measured for each sample to get the mean values. The strain relaxation is performed by a constant-load nanoindentation and set to start when the thermal drift is less than 0.02 nm s^−1^.

### Micro-pillar compression

Hyper-quenched metallic glass samples with a thickness of around 8 μm is prepared for the micro-pillar compression. The micro-pillars with nominal diameters of around 3 μm are prepared by a focused ion beam (FIB) system (JEOL JIB-4600F). The micro-pillars are compressed by a flat-end indenter tip with a diameter of 10 μm at a constant loading rate of 0.1 mN s^−1^ (Shimadzu W201S).

## Electronic supplementary material

Supplementary Information

## Data Availability

All the data that support the findings of this study are available from the corresponding author upon request.
