# Supplementary material for: Spatial heterogeneity as the structure feature for structure–property relationship of metallic glasses
Source: Nat Commun. 2018 Sep 27;9:3965. doi: 10.1038/s41467-018-06476-8 (PMC6160432; doi:10.1038/s41467-018-06476-8)
Supplement: Supplementary file 1 — Supplementary Information [file 41467_2018_6476_MOESM1_ESM.pdf]

SUPPLEMENTARY INFORMATION

Spatial heterogeneity as the structure feature for structure-property relationship of metallic glasses

Zhu *et al.*

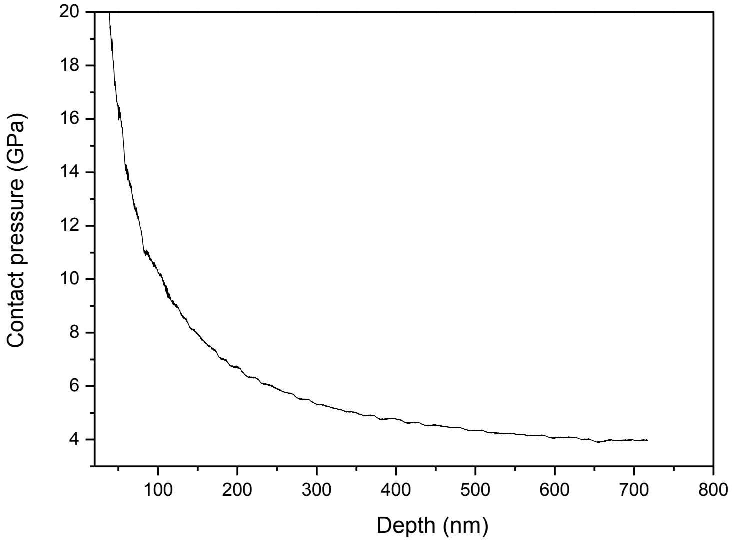

Supplementary Figure 1 | The plot of contact pressure versus the penetration depth derived from the force-depth curve of the hyper-quenched sample in **Figure 2a**. In this study all the hardness (or contact pressure) values are measured at about 600-700 nm penetration depth which is far beyond the depth sensitive region (<300 nm).

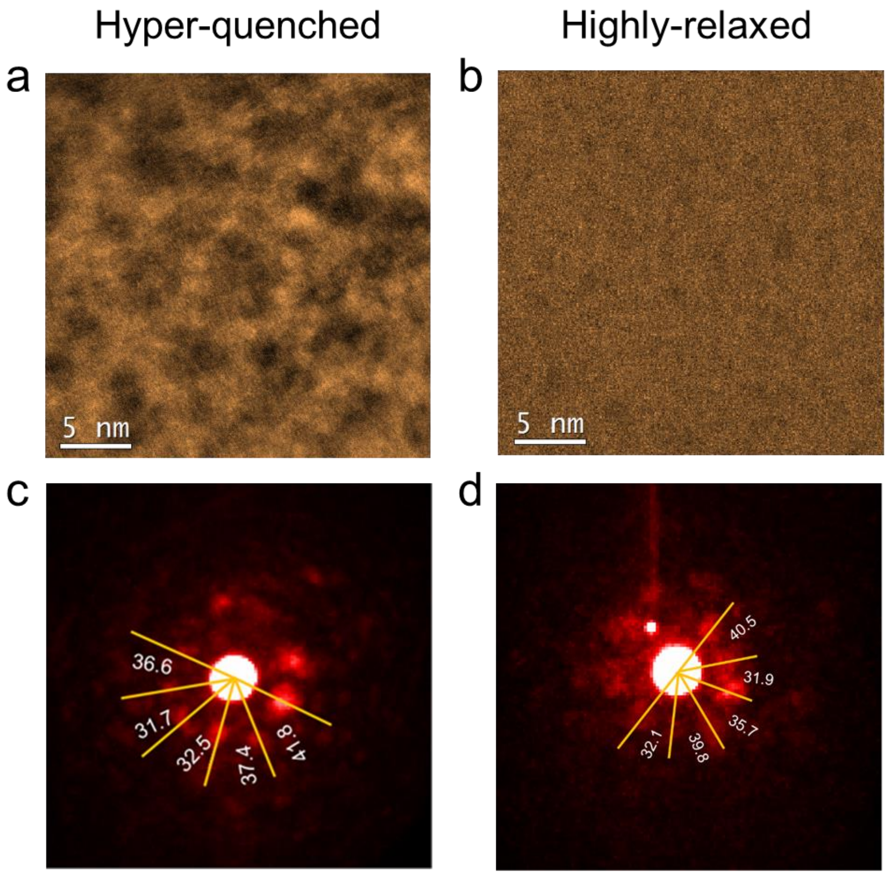

Supplementary Figure 2 | The HAADF-STEM images for (a) the hyper-quenched metallic glass and (b) the highly-relaxed sample. The representative ABED patterns for (c) the hyper-quenched metallic glass and (d) the highly-relaxed sample. Note that the small bright spot in (d) is an artifact induced by the TEM gun.

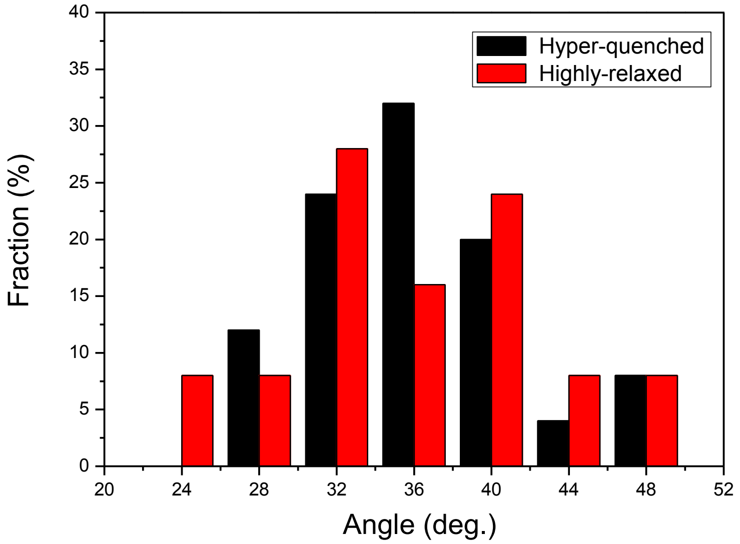

Supplementary Figure 3 | The angular distributions for the five-fold symmetric ABED patterns in the hyper-quenched and the highly-relaxed samples. There are 25 data points for the bar plot.
